# Chidamide plus R‐GDP for relapsed/refractory diffuse large B‐cell lymphoma in patients ineligible for autologous transplantation: A prospective, single‐arm, phase II study

**DOI:** 10.1002/cam4.70142

**Published:** 2024-08-29

**Authors:** Guang‐Liang Chen, Kai Xue, Qunling Zhang, Zu‐guang Xia, Jia Jin, Ran Li, Yizhen Liu, Fangfang Lv, Xiaonan Hong, Xiaoqiu Li, Junning Cao

**Affiliations:** ^1^ Department of Medical Oncology Fudan University Shanghai Cancer Center Shanghai China; ^2^ Department of Oncology Shanghai Medical College Fudan University Shanghai China; ^3^ Shanghai Institute of Hematology, State Key Laboratory of Medical Genomics, National Research Center for Translational Medicine at Shanghai Ruijin Hospital Affiliated to Shanghai Jiao Tong University School of Medicine Shanghai China; ^4^ Department of Hematology, Jiangsu Province Hospital, The First Affiliated Hospital of Nanjing Medical University, Jiangsu Key Lab of Cancer Biomarkers, Prevention and Treatment, Collaborative Innovation Center for Personalized Cancer Medicine Nanjing Medical University Nanjing China; ^5^ Department of Pathology Fudan University Shanghai Cancer Center Shanghai China

**Keywords:** autologous transplantation, chidamide, clinical trial, diffuse large B‐cell lymphoma, epigenetic mechanisms, relapsed/refractory, thrombocytopenia

## Abstract

**Background:**

In relapsed/refractory (R/R) diffuse large B‐cell lymphoma (DLBCL), a negative prognosis is frequently linked to heightened epigenetic heterogeneity. Chidamide, a selective histone deacetylase inhibitor, shows promise as a targeted therapy for R/R DLBCL by targeting abnormal epigenetic changes associated with poor prognosis.

**Methods:**

A cohort of 27 ineligible patients with R/R DLBCL participated in an open — label, single — arm study. Chidamide was administered orally at a dose of 30 mg twice weekly for one week during the induction monotherapy phase. The subsequent combination therapy phase involved oral chidamide at a dose of 20 mg twice weekly for two weeks, followed by a one‐week discontinuation period, in conjunction with intravenous R‐GDP every 21 days.

**Results:**

Among the cohort of 31 patients who underwent screening (median age: 67 years), 27 were ultimately included in the study, with 14 individuals successfully completing six cycles of C‐R‐GDP treatment. The overall best objective response rate was determined to be 79.1% (95% CI: 75.1%–83.3%), comprising a complete response rate of 45.8% (95% CI: 41.6%–49.9%) and a partial response rate of 33.3% (95% CI: 29.3%–37.4%). Within the subgroup of 14 patients who completed the full treatment regimen, the best objective response rate reached 100%, with 71.4% achieving complete response (*n* = 10) and 28.6% achieving partial response (*n* = 4). The median follow‐up period for these patients was 17.0 months, ranging from 3.5 to 55 months. Progression‐free survival was 5.9 months and overall survival was 48.3 months. Anemia was the most common adverse event, affecting all patients. Thrombocytopenia led to treatment interruption or dose reduction in 13 patients. Other common adverse events included hypocalcemia, hyponatremia, and hypokalemia. Three patients experienced grade 3 pneumonitis and one had grade 3 skin rash.

**Conclusions:**

Chidamide combined with R‐GDP is a safe and effective treatment option for patients with R/R DLBCL who are not eligible for autologous stem cell transplantation.

## 1 INTRODUCTION

Although there have been notable advancements in first‐line treatment options, a substantial number of patients diagnosed with diffuse large B‐cell lymphoma (DLBCL) experience relapse or refractory (R/R) disease, resulting in a bleak prognosis ranging from 30% to 40%. Despite the emergence of innovative therapies like chimeric antigen receptor (CAR) T‐cell therapy and bispecific antibodies, salvage chemotherapy remains a vital strategy for managing lymphoma in patients who are not suitable candidates for autologous hematopoietic cell transplantation (auto‐HCT). Finding effective and safe salvage chemotherapy regimens is necessary to address the challenges presented by R/R DLBCL, especially for patients who are not eligible for auto‐HCT.

The molecular pathogenesis of DLBCL is intricately linked to the dysregulation of epigenetic processes.^1^, ^2^, ^3^ Targeting aberrant epigenetic programming is emerging as a promising therapeutic strategy, impeding DLBCL growth, and overcoming resistance to chemotherapy.^1^, ^2^, ^3^, ^4^, ^5^ Chidamide, a selective inhibitor of class I and II histone deacetylases (HDACs) that effectively inhibits tumor cell growth and induces selective apoptosis,^5^ has received approval for the treatment of relapsed or refractory peripheral T‐cell lymphoma.^6^ When combined with rituximab, cyclophosphamide, doxorubicin, vincristine, and oral prednisolone (R‐CHOP), chidamide has been found to be both effective and safe in elderly patients with newly diagnosed DLBCL.^7^ In a subgroup of R/R DLBCL patients, treatment with chidamide as a single agent resulted in an overall response rate (ORR) of 25.0% and a complete response (CR) rate of 15.0%.^2^ Furthermore, chidamide has exhibited the capacity to augment the responsiveness of rituximab^8^, ^9^ and various chemotherapy agents including etoposide, gemcitabine, and cisplatin.^10^, ^11^, ^12^ In totality, chidamide emerges as a robust suppressor of cell survival in DLBCL, presenting itself as a promising therapeutic alternative.^9^, ^13^, ^14^ The exploration of chidamide is warranted to ascertain its efficacy in addressing the urgent and crucial requirement to improve response rates and counteract chemotherapy resistance in salvage chemotherapy for patients with R/R DLBCL.

The R‐GDP regimen, consisting of rituximab, gemcitabine, cisplatin, and dexamethasone administered in 21‐day cycles, has demonstrated comparable efficacy to alternative regimens such as rituximab, dexamethasone, cytarabine, cisplatin (R‐DHAP) or rituximab, ifosfamide, carboplatin, etoposide (R‐ICE).^15^, ^16^, ^17^ Notably, R‐GDP significantly reduces toxicity and presents a more favorable cost profile,^15^, ^18^ positioning it as a feasible and well‐tolerated option for B‐cell non‐Hodgkin lymphoma.^15^, ^16^, ^17^ The objective of this phase II study was to assess the effectiveness and safety of the combination of chidamide with R‐GDP (C‐R‐GDP) in patients with R/R DLBCL who are not suitable candidates for auto‐HCT.

## 2 METHODS

### 2.1 Study design and participants

This open‐label, prospective, single‐arm phase II study was approved by the independent ethics committee of Fudan University Shanghai Cancer Center (no. 1710177‐20) and conducted in accordance with the Declaration of Helsinki and Good Clinical Practice guidelines. Prior to the commencement of any study‐related procedures, all patients provided written informed consent. The study was registered in ClinicalTrials.gov under the identifier NCT03373019.

According to previous research,^15^, ^17^, ^19^ the R‐GDP scheme has demonstrated an observed response rate (ORR) of 60%. This study hypothesizes that the introduction of the C‐R‐GDP scheme will lead to a 15% increase in response rate, resulting in an overall response rate of 75%. To achieve a statistical power of 80% and maintain a one‐way *α* value of 0.05, a sample size of 57 cases, factoring in a 10% dropout rate, has been determined. Therefore, a total of 63 cases are necessary for this study. The Wilson score interval method was used to calculate a confidence interval (CI) for a proportion or rate, such as the ORR, CR rate, or partial response (PR) rate.

The eligibility criteria for this study encompassed individuals aged 18–75 years, with an Eastern Cooperative Oncology Group (ECOG) performance status of 0 or 1. Patients who exhibited adequate organ function but were unwilling or unsuitable for auto‐HCT were included. Additionally, patients diagnosed with de novo DLBCL or transformed lymphoma, as confirmed by biopsy prior to the initiation of the study drug, were classified as R/R cases following at least one prior regimen of multiagent‐containing anthracycline chemotherapy. Important exclusion criteria consisted of previous treatment with any HDAC inhibitor, diagnosed central nervous system lymphoma, and abnormal levels of hepatitis B virus (HBV)‐DNA.

### 2.2 Treatment

Participants were administered chidamide at a dosage of 30 mg twice weekly for a duration of 1 week prior to the initiation of the initial cycle of R‐GDP, denoted as the induction monotherapy phase. For the combination therapy stage, the treatment schedule on a 21‐day cycle basis was as follows: oral chidamide 20 mg twice weekly for 2 weeks and discontinued for one‐week, intravenous rituximab 375 mg/m^2^ on Day 0, intravenous gemcitabine 1000 mg/m^2^ on Days 1 and 8, intravenous dexamethasone 40 mg on Days 1–4, and cisplatin 25 mg/m^2^ on Days 1–3. Delays in dosing were allowed for adverse events (AEs) related to the drug, while adjustments in dosage were not permitted. Patients who did not exhibit rapid disease progression and demonstrated clinical benefit from C‐R‐GDP, along with a stable performance status, were eligible for continued treatment beyond the point of investigator‐assessed disease progression.

### 2.3 Assessments

To assess the effectiveness of treatment, patients underwent evaluation for tumor response based on the Lugano evaluation criteria for malignant lymphoma established in 2015, utilizing fluorodeoxyglucose–positron emission tomography (PET/CT) and spiral computed tomography/magnetic resonance imaging. A baseline evaluation was conducted, followed by assessments every two treatment cycles thereafter. Patients demonstrating tumor remission (CR or PR) were recommended to continue with the initial treatment regimen for a maximum of six cycles. Those without partial tumor remission were advised to discontinue participation in the study. PET/CT imaging was utilized for tumor assessments at baseline and was mandatory to confirm complete remission.

The safety evaluations in this study encompassed the assessment of AEs, clinical laboratory tests, and physical examination, including the evaluation of ECOG performance status. Local laboratory assessments were conducted within 72 hours prior to dosing. Following discontinuation from the study, safety evaluations were planned for the initial follow‐up visit (occurring 35 days after the last dose) and the subsequent follow‐up visit (occurring 80 days after the initial follow‐up visit). Patients were subsequently monitored every 3 months for the presence of ongoing treatment‐related AEs and survival outcomes.

## 3 RESULTS

### 3.1 Patients

**FIGURE 1:**
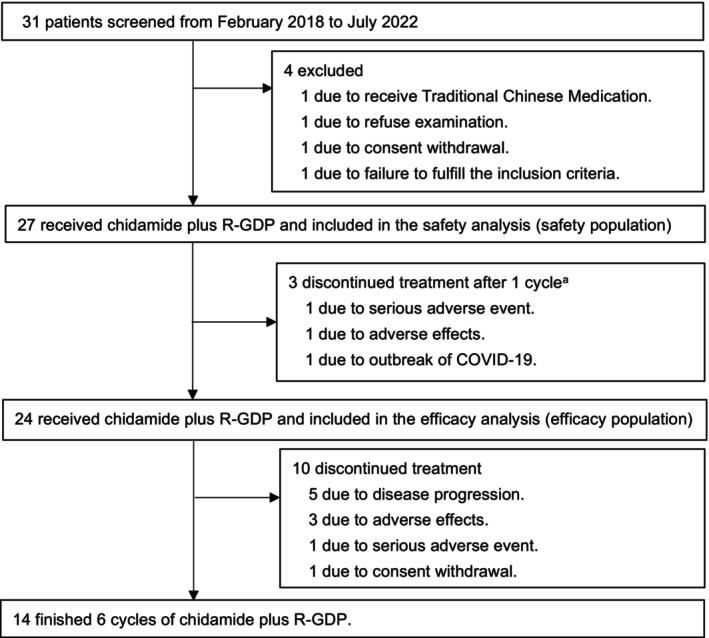
CONSORT (Consolidated Standards of Reporting Trials) diagram. Survival data was collected up until the October 20, 2022 data cut‐off. R‐GDP, rituximab, gemcitabine, dexamethasone, cisplatin. ^a^Radiological post‐baseline response assessment not performed/data unavailable.

Overall, 31 patients were screened for the study from February 2018 to July 2022. Twenty‐seven patients were enrolled, received at least one dose of C‐R‐GDP, and were eligible for safety evaluation (Figure 1). However, three patients who discontinued treatment after 1 cycle were excluded from further tumor response assessment. Twenty‐four patients had performed tumor response assessment and were defined as the efficacy population. Of those, 14 patients had completed six cycles of C‐R‐GDP, while 10 patients had discontinued treatment. In total, 13 patients (13/27, 48.1%) required treatment discontinuation, with AEs (6/27, 22.2%) and disease progression (5/27, 18.5%) being the primary causes (Figure 1). Since low patient recruitment, this study was closed prematurely.

The study provides an overview of the baseline characteristics of the patients, as outlined in Table 1. The median age at enrollment was 67 years, with a range of 51–74 years, and the majority of patients were over the age of 65. The gender distribution was nearly equal between male and female patients. Approximately half of the patients displayed elevated levels of lactate dehydrogenase. All patients had undergone at least one prior treatment with R‐CHOP or a comparable chemoimmunotherapy regimen, with a median time since diagnosis of DLBCL of 14 months. In the study cohort, 23 cases (85.2%) were categorized as stage III/IV, and 15 cases were classified as non‐germinal center B‐cell (GCB) type based on immunohistochemistry analysis. None of the patients displayed double‐ or triple‐hit DLBCL. A total of 20 patients (74.1%) were identified as having R/R DLBCL within 1 year following their last treatment.

**TABLE 1:** Baseline patient and disease characteristics.

	Patients in safety population (*n* = 27)
Age (years)	
Median (range)	67 (51–74)
Age ≥ 65 years, *n* (%)	21 (77.8)
Sex	
Male *n* (%)	13 (48.1)
Female *n* (%)	14 (51.8)
ECOG performance‐status score	
1 *n* (%)	25 (92.5)
≥ 2 *n* (%)	2 (7.4)
Stage III/IV *n* (%)	23 (85.2)
Increased lactate dehydrogenase *n* (%)	12 (44.4)
Cell of origin (by immunohistochemistry), *n* (%)	
GCB	10 (37.0)
Non‐GCB	15 (55.6)
Unknown	2 (7.4)
Patients with transformed lymphoma *n* (%)	
Follicular lymphoma	2 (7.4)
Marginal zone lymphoma	1 (3.7)
Number of previous treatments *n* (%)	
1	25 (92.6)
2	2 (7.4)
First‐line therapy *n* (%)	
CHOP	1 (3.7)
R‐CHOP	19 (70.4)
R‐CHOP+X	7 (25.9)
Type of disease at inclusion *n* (%)	
Relapse^a^	8 (29.6)
Refractory^b^	19 (70.4)
Time since DLBCL diagnosis months (95%CI)	14 (11.0–18.0)
Less than 1 year since last treatment *n* (%)	20 (74.1)

Abbreviations: CI, confidence interval; DLBCL, diffuse large B‐cell lymphoma; ECOG, Eastern Cooperative Oncology Group; GCB, germinal center B cell; R‐CHOP, rituximab, cyclophosphamide, doxorubicin, vincristine, and oral prednisolone.

^a^ Relapse refers to lymphoma, which recurs or develops after a period of complete remission.

^b^ Refractory was defined as patients with stable or progressive lymphoma after first‐line treatment.

### 3.2 Efficacy outcomes

**FIGURE 2:**
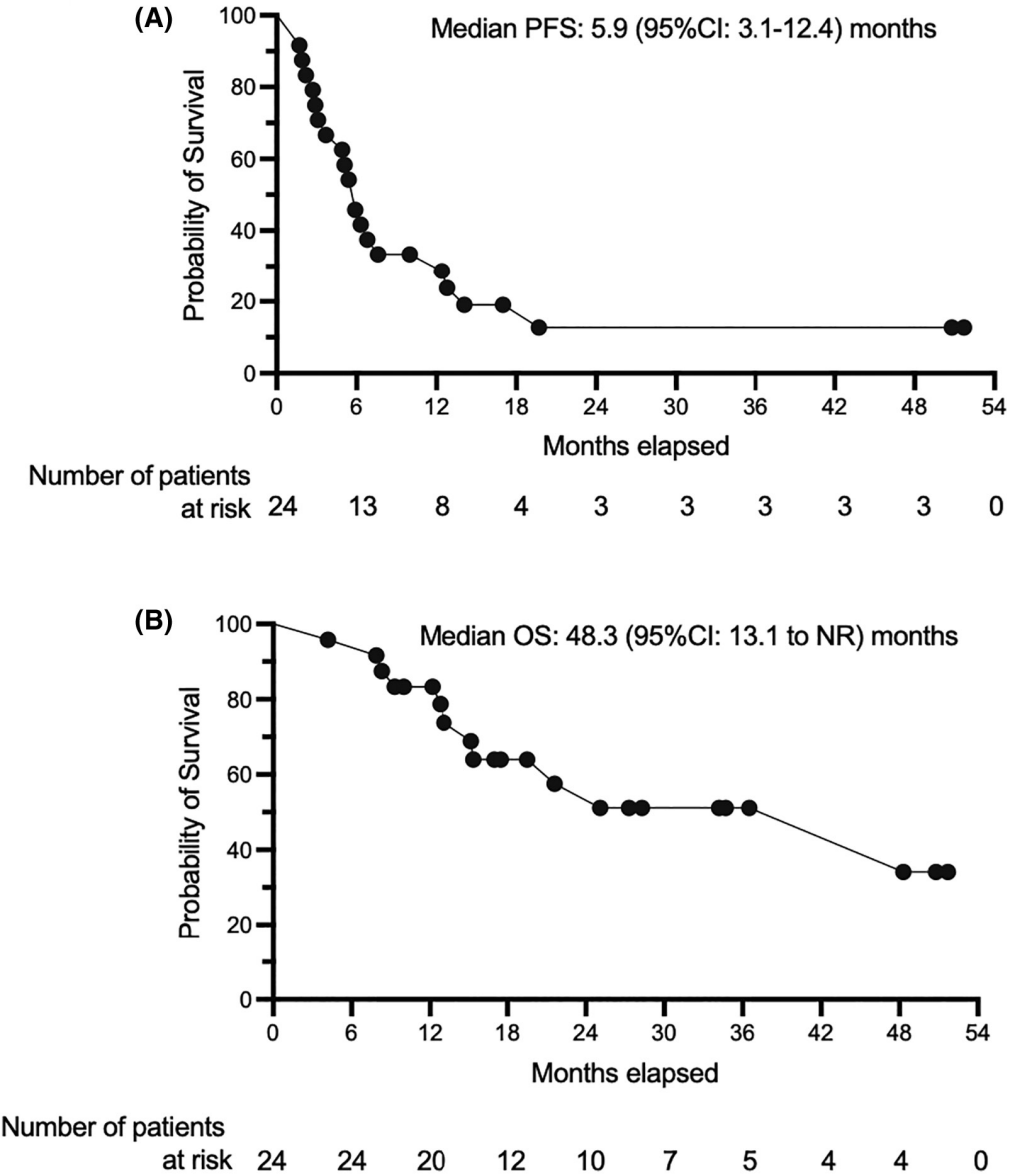
Survival analysis of patients in the efficacy population (*n* = 24). Analyzed by using Kaplan–Meier methodology for progression‐free survival (PFS) (A) and overall survival (OS) (B) censored patients are indicated. NR, not reach.

The median follow‐up period at the data cutoff on October 20, 2022 was 17.0 months, with a range of 3.5 to 55 months. In the efficacy population, the observed ORR rate was 79.1% (19/24; 95% CI, 75.1%–83.3%), the CR rate was 45.8% (11/24; 95% CI, 41.6%–49.9%), and the PR rate was 33.3% (8/24; 95% CI, 29.3%–37.4%) (Table 2). Furthermore, 8.3% of patients (2/24; 95% CI, 4.3%–12.4%) exhibited stable disease. The study demonstrated a disease control rate of 87.5%, with 14 patients completing six cycles of C‐R‐GDP and achieving an ORR of 100%, including a CR rate of 71.4% and a PR rate of 28.6%. Analysis of the efficacy population revealed a median PFS of 5.9 months (95% CI, 3.1–12.4) and a median overall survival (OS) of 48.3 months (95% CI, 13.1–not reach (NR)) (Figure 2). Detailed regimens and treatments administered to patients following the failure of C‐R‐GDP are provided in Table S1.

**TABLE 2:** Investigator assessed best overall response.

	Efficacy population (*n* = 24)
Best objective response *n* (%)	
Complete response	11 (45.8)
Confirmed CR with FDG‐PET^a^	9 (37.5)
Partial response	8 (33.3)
Stable disease	2 (8.3)
Progressive disease	3 (12.5)
Objective response *n* (%) (all patients)	19 (79.1)
Disease control	21 (87.5)

*Note*: Data are number of patients (%) with cut off on October 20, 2022. Objective response: complete + partial responses. Disease control rate: complete + partial responses + stable disease.

Abbreviations: CR, complete response; DLBCL, diffuse large B‐cell lymphoma; PP, per‐protocol.

^a^ FDG‐PET, 18‐fluorodeoxyglucose positron emission tomography.

### 3.3 Safety outcomes

Table 3 illustrates that treatment‐emergent adverse events (TEAEs) of any grade were present in the entirety of the 27 patients under study. The predominant hematologic TEAE of all grades was anemia, affecting each patient (100%), followed by leukopenia (88.9%), and thrombocytopenia (85.2%). Notably, the most prevalent grade 3 or higher adverse event necessitating treatment interruption or dose reduction was thrombocytopenia, impacting 13 patients (48.1%). Subsequently, anemia (37.4%), leukopenia (22.2%), and neutropenia (14.8%) were the subsequent most frequent grade 3 or higher events (see Table 3).

**TABLE 3:** Treatment‐emergent adverse events (any grade) occurring in patients (*n* = 27).

TEAEs^a^ *n* (%)	All grade	Grade ≥3
Hematologic events		
Neutropenia	22 (81.5)	4 (14.8)
Anemia	27 (100)	10 (37.4)
Thrombocytopenia	23 (85.2)	13 (48.1)
Leukopenia	24 (88.9)	6 (22.2)
Non‐hematologic events		
AST increased	4 (14.8)	0
ALT increased	7 (25.9)	0
Blood bilirubin increased	8 (29.6)	0
Creatinine increased	5 (18.5)	0
Hyponatremia	15 (55.6)	0
Hypokalemia	12 (44.4)	0
Hypocalcemia	16 (59.3)	0
Fatigue	8 (29.6)	0
Pneumonitis	4 (14.8)	3 (11.1)
Skin rash	2 (7.4)	1 (3.7)

*Note*: Data are number of patients (%).

Abbrevaitions: AST, aspartate aminotransferase; ALT, alanine aminotransferase.

^a^ TEAEs according to the Medical Dictionary for Regulatory Activities preferred term. Treatment‐emergent adverse events (TEAE) were defined as any adverse event reported in the following time interval (including the lower and upper limits): date of first administration of study treatment; date of last administration of study treatment +30 days, or if they were related to the study drug.

Among non‐hematologic adverse events, hypocalcemia was observed in 59.3% of patients, while hyponatremia and hypokalemia occurred in 55.6% and 44.4% of patients, respectively. All adverse events related to hepatorenal toxicity and electrolyte imbalance were of grade 1–2. Additionally, pneumonitis was observed in four patients, with three of them experiencing grade ≥3 pneumonitis. Two patients developed a skin rash, with one experiencing a grade 3 rash. Among the cohort of patients who underwent six cycles of C‐R‐GDP treatment, 57.1% (eight patients) necessitated a reduction in chidamide dosage due to severe thrombocytopenia of grade 3 or higher. Serious adverse events (SAEs) were documented in a minority of the patient population, specifically affecting only two individuals (7.4% of the total sample). These events included pneumonitis in one patient (3.7%) and severe nosebleeds in another patient (3.7%). No treatment‐related deaths occurred in this study.

## 4 DISCUSSION

Many patients, particularly those elderly or with significant comorbidities, are ineligible for auto‐HCT, yet there remains a lack of established standardized protocols for their care. In the context of this phase II investigation, we have observed that incorporating chidamide into the treatment regimens of R‐GDP results in a notably high ORR among patients with R/R DLBCL who are ineligible for auto‐HCT, all while maintaining a well‐tolerated safety profile. These favorable outcomes suggest that chidamide holds the potential to address the challenge of refractoriness to platinum derivative‐containing salvage chemotherapy for R/R DLBCL.

In this study, we enrolled refractory (70%) and relapsed (30%) DLBCL patients, with 74.1% relapsing within a year post‐treatment, and 55.6% classified as a non‐GCB subtype. Despite these challenges, we met the primary endpoint of the trial and achieved an impressive 79.1% ORR in R/R DLBCL, comparable to chidamide‐based therapy outcomes,^20^ and an improved response than some established regimens like R‐ICE and R‐DHAP show a 63% response rate but come with notable side effects.^21^ Indeed, Ghio et al.^18^ reported 48.8% ORR in R/R DLBCL patients ineligible for high‐dose therapy using R‐GDP. Notably, dose‐adjusted R‐GDP exhibited an 82.8% ORR for auto‐HCT ineligible elderly relapsed DLBCL patients. However, the patient population in Yamasaki et al.'s study,^22^ predominantly with GCB subtype (80.6%), may explain favorable results. Chidamide, combined with venetoclax, down‐regulates MYC, BCL2, and TP53 expression, inhibiting DLBCL growth.^10^, ^11^ Furthermore, the combination of ibrutinib and low‐dose chidamide demonstrates synergistic antitumor effects in B‐cell lymphoma.^23^, ^24^ Interestingly, single‐agent mocetinostat shows an 18.9% ORR in R/R DLBCL.^25^ These findings highlight the potential advantages of targeting epigenetic mechanisms to overcome drug resistance, not only in the context of rituximab but also with other antitumor agents.

Compared to other alternative regimens, R‐GDP therapy appears as a promising option for R/R DLBCL patients ineligible for auto‐HCT due to its lower toxicity profile.^15^, ^18^ While GDP therapy showed grade 3 and 4 thrombocytopenia rates of 24% and 4%, respectively,^16^ an Italian study reported a lower incidence of grade 3 thrombocytopenia related to GDP‐R, affecting only 8.8% of patients.^18^ In Japan, dose‐adjusted GDP‐R resulted in higher platelet count reductions, with 12.1% and 57.6% experiencing grade 3 and 4 reductions, respectively, and 6% encountering grade 3 or worse hyponatremia, with no treatment‐related fatalities.^22^ Notably, we observed no febrile neutropenia with prophylactic long‐term granulocyte colony‐stimulating factor (G‐CSF) administration. However, severe thrombocytopenia led to treatment discontinuation for 13 patients (48.1%), and cases of pneumonitis and skin rash were consistent with prior reports.^26^, ^27^ These adverse event differences may be linked to selection bias, given the majority of patients are ≥65 years old at diagnosis, prompting consideration for optimizing chidamide doses in combination therapy to enhance tolerability.^28^

Our research has revealed that C‐R‐GDP shows promise as a treatment regimen for patients with R/R DLBCL who are not eligible for auto‐HCT. Despite this, there are other treatment options available for this subgroup of patients.^29^ One such option is Pola‐BR, which has demonstrated significant efficacy with a CR rate of 40%.^30^ However, it is important to note that fatal adverse events, including infections, have been reported in patients receiving Pola‐BR or BR therapy. Moreover, studies have demonstrated that immune checkpoint inhibitors used as a single form of therapy have achieved an ORR of 22%, particularly in patients with primary mediastinal B‐cell lymphoma.^31^, ^32^ The combination of lenalidomide with pembrolizumab did not show notable added benefits.^29^ Conversely, the combination of tafasitamab and lenalidomide has exhibited considerable efficacy, resulting in substantial and lasting responses in patients who are not eligible for auto‐HCT.^33^ Furthermore, the Food and Drug Administration (FDA) has recently granted approval for the use of selinexor in patients with R/R DLBCL who have undergone at least two prior therapies. It is worth noting that the combination therapy of selinexor with R‐GDP has demonstrated a notable overall response rate (ORR) of 67% in the treatment of R/R DLBCL, with manageable and reversible adverse events.^34^ In comparison, lenalidomide and rituximab exhibited an ORR of 33% in patients with R/R DLBCL.^35^ Brentuximab vedotin, a CD30‐targeting agent, is recommended as a treatment option in the second‐line setting for patients with transplant‐ineligible CD30‐positive R/R DLBCL.^36^ Moreover, ongoing investigations into targeted therapies like phosphatidylinositol 3 kinase‐β and ‐δ inhibitors, Bruton's tyrosine kinase inhibitors, and CD20‐targeted agents are expanding treatment options for R/R DLBCL patients ineligible for auto‐HCT.^29^ Recently, the CD3xCD20 bispecific antibody, epcoritamab, demonstrated an impressive ORR of 63.1%, including deep and durable CRs, albeit with 6.4% experiencing immune effector cell–associated neurotoxicity syndrome, including one fatal event.^37^ CAR T‐cell therapy holds promise for R/R DLBCL, but its widespread use is hindered by challenges in managing rapidly progressing cases.^29^ In summary, C‐R‐GDP has the potential to impact clinical practice by providing oncologists with a viable alternative therapeutic strategy for managing this difficult‐to‐treat population, thereby enhancing the overall management and care of patients with R/R DLBCL.

Our study possesses certain limitations that necessitate acknowledgment. First, the sample size of patients included in our single‐center experience was small, potentially compromising the generalizability of our findings. Second, the absence of a reliable biomarker to predict a positive response to chidamide plus R‐GDP therapy impeded our capacity to personalize treatment approaches. In the context of R/R DLBCL, CREBBP inactivation has emerged as a promising potential biomarker for predicting sensitivity to chidamide.^2^ However, the efficacy of combining chidamide with an Aurora kinase A (AURKA) inhibitor requires further validation before incorporation into clinical practice.^2^ Future research will focus on elucidating the role of tumor microenvironment factors and identifying additional genetic markers that may predict response to chidamide‐based therapy. In addition, several factors, including physician preference, cost considerations, the proximity of treatment centers, and even the potential influence of the coronavirus disease 2019 pandemic, may have introduced bias and impacted both therapeutic outcomes and disease assessment. To surmount these limitations, future studies should strive to incorporate larger multicenter cohorts, exploring alternative patient populations, and utilize randomized clinical trials, enabling a more comprehensive evaluation of the treatment's efficacy, and addressing potential confounding factors.

## 5 CONCLUSIONS

The findings of this phase II trial suggest that the combination of chidamide and R‐GDP demonstrates a notable response rate and potential efficacy in DLBCL patients ineligible for auto‐HCT, with a favorable safety profile. Further investigation into the impact of biological factors, such as the tumor microenvironment components or genetic markers, is needed to identify specific subgroups of DLBCL patients who may benefit most from chidamide‐based therapy.

## AUTHOR CONTRIBUTIONS

**Guang‐Liang Chen:** Data curation (lead); formal analysis (lead); investigation (lead); project administration (equal); software (equal); validation (equal); visualization (equal); writing – original draft (lead); writing – review and editing (lead). **Kai Xue:** Conceptualization (equal); data curation (equal); formal analysis (equal); resources (equal); writing – original draft (equal); writing – review and editing (equal). **Qunling Zhang:** Data curation (equal); investigation (equal); resources (equal); validation (equal); writing – review and editing (equal). **Zu‐guang Xia:** Investigation (equal); resources (equal); writing – review and editing (equal). **Jia Jin:** Investigation (equal); resources (equal). **Ran Li:** Investigation (equal); resources (equal); software (equal). **Yizhen Liu:** Investigation (equal); resources (equal). **Fangfang Lv:** Formal analysis (equal); investigation (equal); resources (equal); writing – review and editing (equal). **Xiaonan Hong:** Data curation (equal); formal analysis (equal); project administration (equal); resources (equal); supervision (equal); writing – original draft (equal); writing – review and editing (equal). **Xiaoqiu Li:** Data curation (equal); formal analysis (equal); investigation (equal); resources (equal); writing – review and editing (equal). **Junning Cao:** Conceptualization (equal); formal analysis (equal); investigation (equal); project administration (equal); supervision (equal); validation (equal); writing – original draft (equal); writing – review and editing (equal).

## FUNDING INFORMATION

Scientific Research Project of Shanghai Municipal Health Commission (202040040).

## CONFLICT OF INTEREST STATEMENT

The authors declare that they have no competing interests.

## ETHICS STATEMENT

The research conducted in this study received approval from the independent ethics committee at Fudan University Shanghai Cancer Center (no. 1710177‐20) and was carried out in compliance with the Declaration of Helsinki and Good Clinical Practice guidelines.

## CONSENT

Not applicable.

## Supporting information


Data S1.

## Data Availability

Data sharing is not applicable to this article as no datasets were generated or analyzed during the current study.
