# Supplementary material for: Chidamide plus R‐GDP for relapsed/refractory diffuse large B‐cell lymphoma in patients ineligible for autologous transplantation: A prospective, single‐arm, phase II study
Source: Cancer Med. 2024 Aug 29;13(16):e70142. doi: 10.1002/cam4.70142 (PMC11358697; doi:10.1002/cam4.70142)
Supplement: Supplementary file 1 — Data S1. [file CAM4-13-e70142-s001.docx]

**Supplemental Table** 1: Detailed regimens and treatments received by patients after failure of chidamide with R-GDP (C-R-GDP).

| Patient No. | Regimens and Treatments |
| --- | --- |
| 1 | CEOP, Radiotherapy |
| 2 | HMPL-523 (SYK inhibitor) clinical trial |
| 3 | Radiofrequency ablation of liver lesions |
| 4 | Unknown chemotherapy regimen* |
| 5 | R2 |
| 8 | Unknown* |
| 9 | Unknown* |
| 10 | ICE, ATG-010 (selinexor). |
| 14 | HMPL-689 (PI3Kδ inhibitor) clinical trial |
| 17 | Radiotherapy, R-HD-MTX |
| 21 | Unknown* |
| 23 | BR, Pola-R-GemOx |
| 24 | R-ESHAP |
| 25 | Odronextamab (REGN1979) clinical trial |

* Patient or relatives were unable to provide specific details of the regimen or treatment at follow-up. CEOP, cyclophosphamide, etoposide, prednisolone, vincristine; R2, rituximab and lenalidomide; R-ESHAP, rituximab plus etoposide, methylprednisolone, cytarabine and cisplatin; R-HD-MTX, rituximab plus high-dose methotrexate; ICE, ifosfamide, carboplatin, and etoposide; BR, rituximab plus bendamustine; Pola-GemOx, polatuzumab vedotin, rituximab, gemcitabine, and oxaliplatin.
